# Progress in Surgery

**Published:** 1896-04-25

**Authors:** 


### Progress in Surgery.

### GENERAL SURGERY.

(*Continued from page 43.*)

**Lower Extremity.**—Finotti[26] relates a case of complete and permanent recovery after amputation of the hip for sarcomatous tumour developed in callus after fracture of the shaft of the femur. The patient was 53 years old. An exceptional case of dislocation of the hip attributable to infantile paralysis occurring in the earliest period of life is recorded by Appel.[27] The dislocation was not permanent, and the patient soon learnt to replace the bone. Bramanu resected the upper and back part of the capsule with the hope of keeping the bone in place and thus improving the patient's condition. It was too early yet to say what the result will be. John Lowe[28] describes an extension splint devised by him thirty years ago, which, he says, is serviceable for nearly all fractures of the lower extremity at almost all ages. It is made of a bar of nickel-plated or lacquered iron half an inch thick, rounded on the outer and flattened on the inner side; opposite the foot it is bent at a right angle so as to pass across the sole. Opposite the middle of the sole the metal is thickened, and through an opening in this part a stout hook is passed having a handle, by means of which extension can be made. This hook works with a ratchet and spring catch. The upper portion of the splint is attached by means of two bolts fastened by thumbscrews to a padded splint, which is, in fact, the upper portion of an ordinary long splint. It can be adjusted to splints of various lengths; a perineal band and stirrup of strapping to the leg complete the apparatus. John Poland,[29] in recent numbers of The Hospital, gives an exhaustive *résumé* of the various methods of treatment of fracture of the patella, with and without operative intervention. He insists upon the importance of distinguishing between the different forms and conditions of the lesion, and more especially as regards their causation, and gives his own experience in this matter. J. W. White,[30] of Philadelphia, has also made some remarks in the same connection. This surgeon and W. J. Cant[31] advocate Barker's method of subcutaneous intra-articular suture by silk. Each quote three successful cases. E. M. Cox[32] embodies in a paper in the "Annals of Surgery," December, 1895, the details of his method of operative treatment of patellar fracture by suture with catgut, and the use of salt solution. Secondary suture of the patella, with resection of the quadricep's extensor tendon, has lately been successfully performed by W. C. Spencer,[33] while W. W. Keen[34] has divided this muscle in one case, and chiselled loose the tubercle of the tibia as well in two other cases of old fracture, with a successful result. J. B. Fry[35] lately performed amputation of the thigh under the following circumstances in an ordinary oblique fracture of the tibia and fibula: The injury was complicated with comminuted fracture of the neck of the fibula, and excellent recovery ensued for five weeks; there was then evidence of hæmorrhage and probable injury to the anterior tibial artery, which necessitated removal of the limb. Recovery took place.

**Upper Extremity.**—In an interesting paper on separate acromion process simulating fracture, J. Struthers[36] describes this condition very fully, and at the same time draws a distinction between the appearances met with and those occurring in fracture without displacement. The position in the treatment of elbow-joint fractures has been the subject of recent experiments by Smith.[37] He recommends flexion of the elbow to an angle of 45°, with the forearm in the prone position. H. R. Wharton[38] gives short abstracts of fourteen cases of dislocation of the ulnar nerve at the elbow which he finds recorded. In six of these operative treatment was resorted to. He points out that this is a comparatively rare condition, occurring independently of fractures and dislocations. It may result from direct violence, or from muscular effort, or violent flexion of the arm. The symptoms are pain, tingling in the parts supplied by the ulnar nerve, and a certain amount of disability of the elbow, these becoming less prominent in course of time, the nerve accustoming itself to its new position. When the nerve was sutured in its normal position the result was satisfactory, and the dislocation did not recur. In no case is it recorded that neuritis developed in the nerve as a result of the operative treatment. E. H. Bennett[39] has fully illustrated and given in detail his views on injuries of the thumb. M. Schmidt[40] has for some time been treating successfully all fractures, whether simple or compound, of the fingers or toes by an extension apparatus in which the traction is applied directly to the nail of the injured finger or toe. Two small holes are bored in the free edge of the nail, to which indiarubber tubing is attached, and extension made by means of a wooden splint fastened to the hand and healthy fingers by means of strapping.

[26] Epit. Brit. Med. Jour., Dec. 28, 1895; and Wien. Med. Woch., Nov. 30, 1895. [27] Epit. Brit. Med. Jour., Nov. 9, 1895. [28] Clinical Sketches, Dec. 1895, p. 191. [29] The Hospital, Feb. 15, 1896, pp. 329 and 345. [30] Annals of Surgery, Nov., 1895, p. 661. [31] Quarterly Med. Jour., Oct., 1895, p. 42. [32] Annals of Surgery, Dec. 1895, p. 725. [33] Lancet, Nov. 23, 1895, p. 1,291. [34] Annals of Surgery, Nov. 1895, p. 671. [35] Lancet, Dec. 14, 1895, p. 1,495. [36] Edinburgh Med. Journ., Oct., 1895, p. 289; ibid. April, 1896. [37] Boston Med. and Surg. Jour., vol. cxxxi., No. 16, p. 387. [38] Am. Jour. of Med. Science, Oct., 1895; and Med. Chronicle, Nov., 1895, p. 119. [39] Dublin Jour. of Med. Science, Dec., 1895, p. 473. [40] N.Y. Med. Jour., Nov. 30, 1895, p. 702; and Medical Week, Oct. 18, 1895.

